# Fire Protection at the Royal Portsmouth Hospital

**Published:** 1904-10-08

**Authors:** 


### FIRE PROTECTION AT THE ROYAL PORTSMOUTH HOSPITAL.

In order to further secure the safety of the patients in case of fire, the whole of the fire appliances have been lately overhauled at the Royal Portsmouth Hospital. New chemical engines have been placed in the wards and corridors, and a length of hose with a special turncock placed in the children's ward in the old and most vulnerable part of the hospital. In addition a new outlet has been constructed in the last new wing, and iron ladders fixed to the walls of the Nurses' Home and the servants' dormitories.

Last week the new arrangements were thoroughly tested by the Borough Fire Brigade under the superintendence of the Chief Constable. The Brigade, receiving a surprise call, underwent a series of drills. The horsed fire escape was run up to the highest point of the buildings, jumping sheets were brought into use, and at a height of 20 feet some of the nurses tried a supposed hasty retreat by this means. The nurses and porters were shown the precautions usually taken in cases of fire for the resuscitation of injured persons. The new iron ladders were put to actual test, and the chemical engines quickly extinguished a large fire of wood shavings placed in the grounds. The Fire Brigade have kindly offered to repeat their drill quarterly, and this should prove of very valuable assistance should so unfortunate an occurrence as a fire ever happen.

